# Extended range proteomic analysis of blood plasma from schizophrenia patients

**DOI:** 10.3389/fmolb.2024.1483933

**Published:** 2024-11-21

**Authors:** Denis V. Petrovskiy, Tatiana V. Butkova, Kirill S. Nikolsky, Arthur T. Kopylov, Valeriya I. Nakhod, Liudmila I. Kulikova, Kristina A. Malsagova, Nikolai D. Kibrik, Vladimir R. Rudnev, Alexander A. Izotov, Anna L. Kaysheva

**Affiliations:** ^1^ Laboratory of Structural Proteomics, Institute of Biomedical Chemistry, Moscow, Russia; ^2^ Moscow Research Institute of Psychiatry – Branch of the V. Serbsky National Medical Research Centre of Psy-chiatry and Narcology of the Ministry of Health of the Russian Federation, Department of Sexology, Moscow, Russia

**Keywords:** proteins, *de novo* identification, PowerNovo, schizophrenia, serum, post-translational modifications, DDA-MS

## Abstract

**Introduction:**

The high prevalence of schizophrenia worldwide makes it necessary to proceed from subjective assessment of patient’s clinical symptoms in diagnosis making to searching for circulating blood biomarkers. On the one hand, searching for molecular markers and targets for therapeutics will make it possible to refine and detail the molecular mechanisms of pathology development, while on the other hand, it will offer new opportunities for elaborating novel approaches to disease diagnosis and enhance efficacy and timeliness of drug therapy.

**Methods:**

In this study, we performed an extended-range proteomic analysis of plasma samples collected from 48 study subjects with confirmed diagnosis of schizophrenia and 50 healthy volunteers. The high-resolution tandem mass spectra recorded in the data-dependent acquisition mode were analyzed using the MaxQuant algorithm for the library of known protein sequences and the PowerNovo algorithm for *de novo* protein sequencing.

**Results:**

It was demonstrated that both strategies show similar results for high-abundance proteins (≥1 μg/mL). For mid-abundance (10 ng/mL – 1 μg/mL) and low-abundance (<10 ng/mL) proteins, the results obtained by the two search strategies complement each other.

**Discussion:**

Group-specific proteins for the samples of schizophrenia patients were identified, presumably being involved in synaptic plasticity, angiogenesis, transcriptional regulation, protein stabilization and degradation.

## 1 Introduction

Schizophrenia is one of the causes of disability globally (Howes et al., 2024). It affects approximately 24 million people, or one out of 300 people (0.32%) worldwide. Among the adult population, its prevalence is one out of 222 people (0.45%). Disease manifestation most commonly occurs in late adolescence and at an age of 20–30 years (Schizophrenia, 2022; Solmi et al., 2023).

Schizophrenia is accompanied by significant stress and challenges in personal relationships, family life, social contacts, education, professional life, and other important life spheres. Individuals with schizophrenia are two to three times more likely to die early compared to the general population because of physical illnesses such as cardiovascular, metabolic, and infectious diseases (Schizophrenia, 2022).

The exact cause of schizophrenia is unknown. It is believed that schizophrenia may result from an interplay between a number of genetic and environmental factors. Psychosocial factors may also affect the onset and the course of this disease (Tandon et al., 2024). Schizophrenia is a condition where the genetic background has a significant effect on the potential manifestation of the disease. Genetic risk factors presumably involve thousands of common genetic variants, each having a small impact on individual risk, and many rare genetic variants having a stronger individual effect on the overall risk (Tandon et al., 2024). Despite the intense research into the molecular foundations of the disease pathogenesis, including the attempts to identify genetic (Owen et al., 2016) and protein markers (Goldsmith et al., 2018) as well as neurotransmitters (Luvsannyam et al., 2022; Hany et al., 2024), these findings have not been used in medicine yet. Like many other neuropsychiatric disorders, schizophrenia is diagnosed clinically based on the presence or absence of clinical symptoms (Comes et al., 2018) where the psychiatrist’s personal experience plays a crucial role. The fact that needs to be taken into account is that the symptoms, especially during the early stages of the disease, may be episodic and transient, which poses a serious challenge for timely differential diagnosis (Lin et al., 2022). Early detection of schizophrenia is important, since early manifestations of the disease are better correctable and stable remission can be achieved. Furthermore, mental illnesses have a greater economic burden compared to chronic somatic diseases such as cancer or diabetes (Trautmann et al., 2016). Therefore, early laboratory diagnosis and appropriate therapy minimize the expenses.

The key objective of proteomics is to search for objective circulating markers of diseases. Proteomics involves the analysis of proteins in a system at a particular instant under certain conditions and is indicative of pathophysiological processes occurring in the body. Proteomic analysis is among the informative omics approaches, which allows one to identify group-specific proteins, differentially expressed proteins, and protein isoforms (proteoforms). These data are extremely important for creating the digital signature of patient’s biological sample, revealing the features of molecular composition, and identifying the signaling pathways of a pathology. Several proteomic analysis strategies are currently available, which can be applied in one study either as complementary techniques or separately. The first strategy is protein identification using proteomic search engines and libraries of protein sequences. The second strategy is *de novo* peptide sequencing without using libraries. In it, identification of a peptide (a contig) is performed by a neural network that predicts the peptide sequence in accordance with the experimental spectra. The third strategy is identifying peptides and proteins using spectral libraries (the so-called “reference” spectra).

Protein identification strategies in biological samples of complex composition are so diverse because the “width” of the proteomes of organisms still remains unclear. Each protein in the analyzed sample can be represented by several proteoforms caused by single-point amino acid substitutions, post-translational modifications (PTMs) of proteins, and alternative splicing (Ponomarenko et al., 2016). Although the human proteome is encoded by ∼ 20,000 genes, it is populated by several hundred thousand to several million proteins and their proteoforms according to different predictions (Aebersold et al., 2018). Identifying proteoforms and their exact amino acid sequence, as well as studying their functions and properties, are important objectives for understanding the molecular foundations of pathology development.

In this study, we conducted an extended-range proteomic analysis of plasma samples from schizophrenia patients (n = 48) and conditionally healthy volunteers (n = 50) for recording tandem mass spectra in the data-dependent acquisition (DDA) mode. Two identification strategies were used: the MaxQuant approach (Tyanova et al., 2016) enabling interpretation of mass spectra compared to the human protein sequence database and the PowerNovo tool for *de novo* protein sequencing (Petrovskiy et al., 2024). On the one hand, comparative analysis using two strategies of peptide MS/MS assignment broadens the search field, while increasing the fidelity of the revealed identifications on the other hand. An important result of the analysis is searching for new sequences: candidate proteins or proteoforms that have not been annotated in the human protein database.

## 2 Materials and methods

### 2.1 Subjects

We enrolled totally n = 98 subjects for this study among which n = 48 subjects were being schizophrenic subjects who were accepted for inpatient, n = 50 were healthy volunteers aligned by anthropometric data (age, genders ration, BMI) (Table 1).

**TABLE 1:** The sociodemographic characteristics of study participants.

Parameter		Males	Females
General data	Number of participants	25	23
	Median age, years	28 ± 6	25 ± 6
	BMI	23 ± 2.5	21.1 ± 5.4
	History of TBI	8	5
Education	Higher education and incomplete higher education	15	10
	Secondary education	10	13
Marital status	Single, divorced, widowed	24	16
	Married (living with partner)	1	7
Children presence	No	24	16
	Yes	1	7

The general inclusion criteria were as follows.

– the patient was aged >18 years;
– the patient had signs of schizophrenia, schizotypal personality disorder and schizoaffective disorder (ICD-10 codes F20.0, F20.2, F20.8, F21.8, F25.1, and F25.2);
– the patient was diagnosed with schizophrenia or schizophrenia spectrum disorder and met the criteria of the first psychotic episode: disease duration up to 5 years; no more than three episodes;
– the patient provided voluntary informed consent for psychiatric assessment.

The non-inclusion criteria were as follows: a concomitant decompensated somatic or neurological pathology (acute or severe chronic somatic and/or infectious disease), neuroinfections, epilepsy, organic damage to the CNS of any etiology, administration of medications on a regular basis; psychoactive substance and alcohol abuse; pregnant or breastfeeding women. Patients’ neurological and somatic status was verified by conducting expert examination by physicians of related medical disciplines.

An analysis of the anthropometric data showed that the median age of male and female patients was 28 ± 6 and 25 ± 6 years, respectively. Body mass index (BMI) was 23 ± 2.5 in males and 21.1 ± 5.4 in females. This index demonstrated that males either had normal body mass or were overweight, while females had normal body mass or were underweight or overweight. Patient-reported history of traumatic brain injury (TBI) was documented in eight males and five females. Table 1 shows that patients with higher and incomplete higher education prevailed (n = 15) among males, while among females, those with secondary education prevailed (n = 13). It is fair to assume that this result is related to the fact that women tend to make a family at an earlier age than men. An analysis of the marital status demonstrated that women were more likely to be in a family relationship, unlike men who preferred to lead an independent lifestyle. Most men had no permanent partner and one had a family relationship. For women, this ratio was 70% and 30%, respectively. The presence of children also correlated with the marital status: males had no children, while one-third of females had children at the time of the study.

The study design was approved by the local Ethical Committee of the Alexeev N.A. First Clinics of Mental Health, (Moscow; AXM-EH2019-R017.G12 issued on 15 February 2019; AXM-EH2020-R004.Y04 issued on 4 March 2020). All handlings and use of material were provided according to the WMA Declaration of Helsinki on Ethical Principles for Medical Research Involving Human Subjects (revision Fortaleza, 2013). All the participants were aware of the research purpose. Informed consent was obtained from all participants of the control group and informed consent was obtained from a parent and/or legal guardian of participants of the assay group.

### 2.2 Sample handling and LC-MS/MS analysis

The procedures of preliminary preparation of biological samples and LC-MS/MS analysis were thoroughly described in our earlier study (Kopylov et al., 2023). The datasets generated and analyzed during the current study are available in the PRIDE (Perez-Riverol et al., 2021) (Proteome Exchange) repository under registered ID: PXD035863 (ProteomeXchange dataset). Proteomic analysis was performed on an ultra-high-resolution Orbitrap Fusion mass spectrometer equipped with a nanoflow Ultimate 3000 UPLC system (Thermo Fisher, Germany) in the DDA mode (Kopylov et al., 2023).

### 2.3 Bioinformatic analysis

The obtained raw mass spectrometry data were analyzed using the Max Quant software (version 1.6.13) (Wichmann et al., 2019). The results are presented in PXD035863 (ProteomeXchange dataset). *De novo* analysis was conducted using the PowerNovo tool. Results of the analysis are available on FigShare (https://doi.org/10.6084/m9.figshare.26790520.v1). The key parameters were: a) contigs were constructed using de Bruijn assembler ALPS (k-mer = 8). b) The mapped contigs were aligned to the reference protein sequence using the BLAST-like algorithm. We only used contigs with at least 75% identity (Petrovskiy et al., 2024).

**FIGURE 1:**
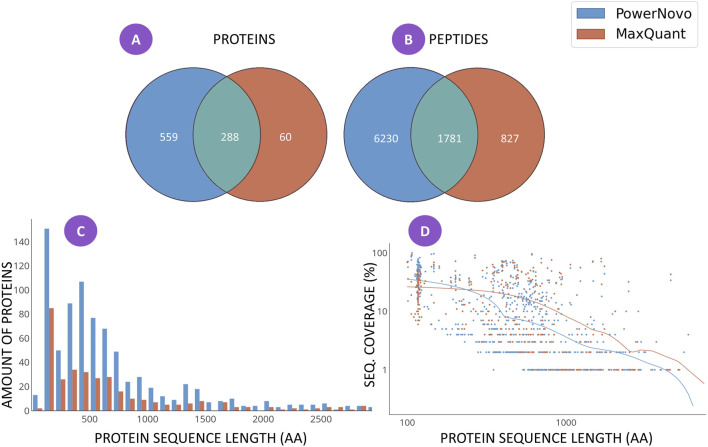
Matching identifications between the two strategies, which were detected in at least ten plasma samples in both comparison groups (schizophrenia patients and groups of conventionally healthy volunteers): the number of identifications of proteins **(A)** and peptides **(B)**; the distribution histogram for the number of protein identifications depending on sequence length for each search strategy **(C)**; and **(D)** the scatterplot of coverage of the amino acid sequence of a protein with identifications depending on sequence length for each search strategy. The comprehensive list of identifications is provided in Supplementary Tables S1, S2.

**FIGURE 2:**
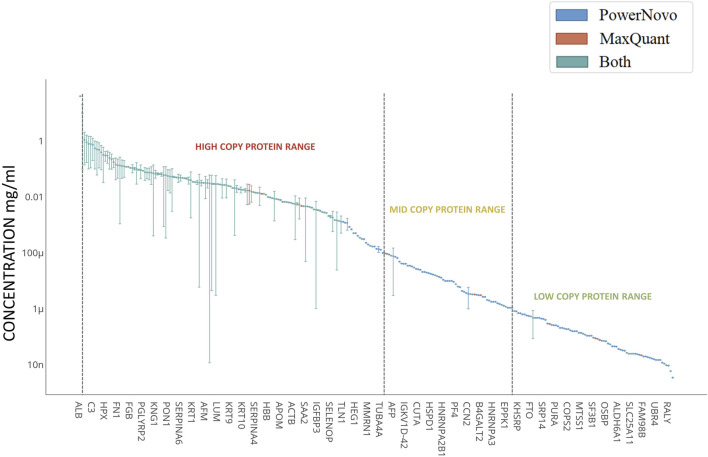
Concentrations of detected circulating proteins. Plasma proteins shown in the left-hand-side can be classified as high-abundance proteins (>1 μg/mL); proteins clustered in the central part correspond to mid-abundance proteins (from 1 μg/mL to 10 ng/mL); and proteins clustered in the right-hand side correspond to low-abundance proteins (<10 ng/mL). *X*-axis shows the genes corresponding to the identified proteins. The concentration regions for proteins are shown with arrows. Proteins identified using both strategies (Common) are shown in blue; proteins identified using the MaxQuant proteomics software are shown in red; and those identified using the PowerNovo tool, in green. The comprehensive list of proteins is presented in Supplementary Table S2.

A comparative analysis of the results of identifications using two strategies was performed using Python scripts employing the Pandas data analysis library (https://pandas.pydata.org/). The data on peptides identified using both strategies were normalized to the general structure, and sequence clustering of peptides and contigs was then conducted using the Usearch engine (https://www.drive5.com/usearch/). The cluster united sequences with ≥95% similarity, as well as matched sequences of different lengths (*de novo* contigs, the missed trypsin cleavage site). The diagrams shown in Figures 1, 2 were plotted using the Plotly library (https://plotly.com/python/). Enrichment with new information about the detected proteins was performed using the library designed by us (available at GitHub: https://github.com/protdb/uniprot_meta_tool). The reference values of plasma concentrations of proteins were taken from API ProteinAtlas (Uhlén et al., 2015). Three-dimensional structures for Table 3 were analyzed using the PyMol software (https://www.pymol.org/).

## 3 Results

### 3.1 De novo assembly enriches the results of protein analysis of the sequence database search engine

At the first stage of the study, we conducted a comparative analysis of the proteome “width” using the MaxQuant sequence database search engine and the PowerNovo tool for *de novo* protein sequencing (Figure 1).

Figure 1 shows the results on the “width” of identifications revealed using the MaxQuant and PowerNovo algorithms. A total of 907 proteins (Figure 1A) were detected. A total of 348 proteins were identified using the MaxQuant proteomic software package, most of which were also detected by the PowerNovo algorithm. The use of PowerNovo also additionally revealed 559 proteins. A similar situation was detected by analyzing the number of identified peptide sequences (Figure 1B). A total of 8,800 peptide sequences were detected using the MaxQuant and PowerNovo algorithms. Of those, 1781 peptides were identified by both algorithms. One can see that these tools increase the number of proteins and peptides identified.

Figure 1C shows the distribution of the number of protein identifications depending on protein length. For both search strategies, the largest number of proteins was detected for the range of 100–1,000 amino acid residues. One can see in Figure 1C that the number of proteins identified using the PowerNovo approach is substantially larger than that identified using the MaxQuant approach for the specified range. The figure indicates that *de novo* sequencing allows one to detect more proteins over the entire range of sequence lengths. Generally comparable results were obtained by comparing the two identification strategies in terms of protein sequence coverage (Figure 1D). The distribution of amino acid sequence coverage as a function of protein length was demonstrated. One can see that both strategies are characterized by virtually equal efficiency of sequence identification coverage for small and medium-sized proteins up to 1,000 amino-acid residue (aa) long. For large proteins (>1,000 aa long), the MaxQuant algorithm is more efficient in terms of protein sequence coverage.

Both strategies were also highly matched for such parameter as analysis depth. Figure 2 shows proteins detected in plasma samples in both comparison groups according to the Protein Atlas data (Uhlén et al., 2015). Proteins detected in plasma are nominally ranked into three groups according to their abundance: high-abundance (>1 μg/mL), mid-abundance (from 1 μg/mL to 10 ng/mL), and low-abundance (<10 ng/mL) ones. A total of 212 identifications were made in the high-abundance protein zone. Most of the proteins (∼85%) were detected using both strategies. Extensive groups of plasma proteins engaged in complement activation (GO:0006956, n = 17), acute inflammatory response (GO:0002526, n = 15), humoral immune response (GO:0006959, n = 22), lipid metabolic processes (GO:0006629, n = 16), etc. were detected in this zone. As the protein abundance level decreases and one proceeds to the zone of mid-abundance proteins (n = 179), the number of identifications made using both strategies declines, being approximately one-third. Proteins involved in cell-cell adhesion (GO:0098609, n = 17) and cell migration (GO:0016477, n = 20), regulation of protein metabolic processes (GO:0051246, n = 43), and transport (GO:0006810, n = 55) according to String DB version 12.0 (Szklarczyk et al., 2023) refer to this zone. The zone of low-abundance proteins is shown in Figure 2 with some limitations, since quantitative contents of more than 500 proteins in plasma have not been annotated yet. The comprehensive list of proteins is provided in Supplementary Table S2. The use of both strategies allows one to extend protein identification ranges. It is noteworthy that low-abundance proteins are especially promising for biomedicine.

### 3.2 Group-specific proteins

Table 2 provides the list and description of proteins identified in plasma proteins mostly in schizophrenia patients. Group-specific proteins are involved in transcriptional regulation, cation binding and transport, development of the nervous system, signal transduction, etc. The selected protein search strategies complement and verify the results obtained by each of them. Thus, some proteins were detected by one of the strategies, but there also were proteins successfully detected by both strategies. According to their location, proteins can be classified as cellular, membrane, and secretory ones. In the UniProt knowledgebase (UniProtKB), proteins listed in Table 2 have a status “evidence at protein level”; blood concentrations were annotated in ProteinAtlas for some proteins: Ephrin-B3 (11 ng/L), VEGF receptor 1 (87 ng/L), Glypican-4 (190 ng/L), Immunoglobulin heavy variable 1–45 (180 μg/L), and SAA2 (50 μg/L). The proteins were annotated in PeptideAtlas in the experiments for blood plasma. According to the Blast data, the number of detected proteotypic peptides (PTPs) is at least one (a criterion).

**TABLE 2:** Proteins detected predominantly in plasma samples of schizophrenia patients.

UniProt ID	Name	Mw, kDa	Lengh, aa	Gene	Localization	Biological processes	Cnt	Sz	PowerNovo (аа)	MaxQuant (аа)	PeptideAtlas	ProteinAtlas	PTPs
Q96MX3	Zinc finger protein 48	68	618	ZNF48	nucleus	Transcription regulation	5	31	+ (20)	–	Plasma	–	2
Q96PN7	Transcriptional-regulating factor 1	132	1,200	TRERF1	nucleus		0	15	–	+ (13)	Plasma	–	1
Q14207	Protein NPAT	154	1,427	NPAT	cytoplasm		1	15	–	+ (18)	Plasma	–	1
P0DJI9	Serum amyloid A-2 protein	14	122	SAA2	high-density lipoprotein particle	Acute phase	3	20	+ (69)	+ (33)	Plasma	50 μg/L	4
Q86UX2	Inter-alpha-trypsin inhibitor heavy chain H5	105	942	ITIH5	extracellular region	Hyaluronan metabolic process	2	10	+ (13)	–	Plasma	–	1
P78352	Disks large homolog 4	80	724	DLG4	synapse		1	9	–	+ (33)	Plasma	–	2
Q15768	Ephrin-B3	36	340	EFNB3	synapse	Neurogenesis	0	17	+ (18)	+ (12)	Plasma	11 ng/L	3
P17948	VEGF receptor 1	151	1,338	FLT1	extracellular space	Angiogenesis	0	13	+ (9)	+ (31)	Plasma	87 ng/L	2
Q96JB5	CDK5 regulatory subunit-associated protein 3	60	506	CDK5RAP3	membrane	Brain development	3	19	–	+ (27)	Plasma	–	1
Q15283	Ras GTPase-activating protein 2	97	850	RASA2	cytosol	Signal transduction	2	10	+ (19)	–	Plasma	–	3
Q14654	ATP-sensitive inward rectifier potassium channel 11	44	390	KCNJ11	synapse	Potassium transport	3	16	–	+ (11)	Cancer cell lines, blood	–	1
O00507	Probable ubiquitin carboxyl-terminal hydrolase FAF-Y	291	2,555	USP9Y	cytoplasm	Ubl conjugation pathway	3	15	+ (20)	+ (19)	Plasma	–	1
Q15751	Probable E3 ubiquitin-protein ligase HERC1	532	4,861	HERC1	cytosol		3	16	+ (16)	+ (17)	Plasma	–	3
Q96LB3	Intraflagellar transport protein 74 homolog	69	600	IFT74	nucleus		2	15	–	+ (27)	Plasma	–	1
Q99973	Telomerase protein component 1	290	2,627	TEP1	cytoplasm	Telomere maintenance via recombination	0	14	–	+ (15)	Plasma	–	1
A0A0J9YWN2	Immunoglobulin heavy joining 2	2	18	IGHJ2	extracellular region	Immune response	0	15	+ (12)	–	Plasma	–	3
A0A0A0MS14	Immunoglobulin heavy variable 1-45	14	117	IGHV1-45	extracellular region		0	16	+ (12)	–	Plasma	380 μg/L	1
O75487	Glypican-4	62	556	GPC4	synapse	Regulation of neurotransmitter receptor localization	0	22	–	+	Plasma	190 ng/L	1

Lengh, aa–protein length expressed as amino acid residues; Localization–localization in the cell; Cnt and Sz–the number of conditionally healthy study subjects and schizophrenia patients in whose samples the protein was detected; PowerNovo and MaxQuant (aa) – identification strategies used to detect the protein; PeptideAtlas–observed in experiments (https://peptideatlas.org); ProteinAtlas–blood concentration of protein in the experiments (https://www.proteinatlas.org/, Human_2024–01); PTPs–the number of detected proteotypic peptides according to the BLAST data (https://www.uniprot.org/blast).

### 3.3 Putative proteoforms

Detecting disease-specific proteoforms is crucial for understanding the nature of schizophrenia. Post-translational modifications (PTMs) often affect protein function. The results of searching for phosphorylated proteoforms, including those carrying phosphorylated serine (SEP), phosphorylated threonine (TPO), and phosphorylated tyrosine (PTR), are summarized in Table 3, which lists important biological and structural data about the proteoforms. In this study, we detected nine putative proteoforms. Proteins are mostly extracellular in terms of their localization; they are involved in the development and functioning of the nervous system and protein metabolism. In structural respect, the experimental model (PDB) was obtained only for LRIG1 protein; the performance of the predicted models of proteins (AlphFold) is generally >80% and is satisfactory (the AFcfd parameter). In some proteins, the phosphorylation site has poor spatial resolution; in other proteins, it is located in the organized secondary structure elements (α-helix or β-strand). One can see that phosphorylation increases the solvent-accessible surface area (K_SASA_ < 1) for all the proteins.

**TABLE 3:** Structural characterization of the phosphorylation sites in proteins with annotated 3D structures according to the PDB and AlphaFold databases.

Uniprot ID	Name	Mw, kDa	Lengh, aa	Gene	Localization	Biological processes	Sequence	Sz	Secondary structure	Source	Structure	Ksasa	AFcfd	PeptideAtlas	ProteinAtlas
O43166	Signal-induced proliferation-associated 1-like protein 1	200	1804	SIPA1L1	postsynaptic density	Regulation of axonogenesis	SSQEIETSSCLDSLS[SEP]K	6	n/d	AF	1	0.89	37.1 ± 27.3	Plasma	–
P35080	Profilin-2	15	140	PFN2	Exosome, synapse	Regulation of synaptic vesicle exocytosis	[SEP]QGGEPT[PTR]NVAVGRAGRVLVFVMGK	4	β-hairpin	AF	1	0.36	96.9 ± 5.8	Plasma	19 ng/L
P83859	Orexigenic neuropeptide QRFP	15	136	QRFP	extracellular region	neuropeptide signaling pathway	A[SEP]QPQALLVIARGLQT[SEP]GREHAGCR	5	α-helix	AF	1	0.94	56.7 ± 10.9	n/d	–
Q5JSJ4	Integrator complex subunit 6-like	97	861	INTS6L	integrator complex	snRNA 3′-end processing	[TPO]LPPYYLLTK	12	α-helix	AF	1	0.36	79.8 ± 23.9	Plasma	–
Q6PDB4	Zinc finger protein 880	67	577	ZNF880	nucleus	Regulation of transcription by RNA polymerase II	NQLGLTFQLHL[SEP]ELQLFQAERNISGCK	4	n/d	AF	1	0.96	81.9 ± 25.1	Plasma	–
Q96SE7	Zinc finger protein 347	96	839	ZNF347			NQLGLSLQ[SEP]HLPELQLFQYEGK	4	n/d	AF	1	0.94	80.5 ± 23.8	Plasma	–
Q96JA1	Leucine-rich repeats and immunoglobulin-like domains protein 1	119	1,093	LRIG1	extracellular space	Innervation	LTDGAFWGL[SEP]K	5	n/d	PDB	1	0.27	–	Plasma	78 ng/L
Q99961	Endophilin-A2	42	368	SH3GL1	synapse	Central nervous system development	AKLTMLNTV[SEP]KIR	5	α-helix	AF	1	0.64	92.9 ± 20.5	Plasma	390 ng/L
Q9H672	Ankyrin repeat and SOCS box protein 7	36	318	ASB7	cytosol	Protein ubiquitination	VFLEHGADP[TPO]VK	5	α-helix	AF	1	0.25	95.9 ± 9.7	Blood cells	–

PTMs were detected using the MaxQuant algorithm; Number of structures–the 3D protein structures annotated in open sources PDB (https://rcsb.org) and AlphaFold (https://alphafold.ebi.ac.uk/); N_pdb/AlfaFolf_–the number of structures annotated in PDB/AlphaFold with this peptide; the coefficient of changes in solvent-accessible surface area, K_SASA_–the ratio between the SASA values of the peptide in intact and phosphorylated proteins.

The search area is extended using the *de novo* protein sequencing strategy, which is not limited by completeness and quality of the protein sequence database. On the one hand, it allows one to detect amino acid sequences (contigs) not annotated in protein databases, while making it possible to identify noncanonical proteoforms on the other hand. In our study, we used the PowerNovo strategy to detect contigs that are found in blood samples of schizophrenia patients and cannot be confidently matched with human proteins or orthologs of other organisms (Blast, https://www.uniprot.org/blast). Table 4 lists the most vivid examples.

**TABLE 4:** Amino acid sequences (contigs at least 10 aa long) most frequently detected in plasma samples of schizophrenia patients. (significant.csv).

Contig siq	Tryptic peptide	Mw, kDa	Lengh, aa	Aliphatic index	Blast (origin)	Sz	Cnt	Total
GLYGGPSYGYGAPTSQR	+	1.7	17	29	88% Lysyl endopeptidase (*Pseudomonas aeruginosa*)	14	1	15
RPHSMSALEVDEGSGSNPGS	–	2	20	39	–	11	1	12
EGTPEAPTAPTDECKLR	+	2	17	35	75% Serotransferrin (*Homo sapiens*)	9	1	10
YGEEMADHCPS	–	1	11	9	–	19	3	22
SLMVNLENVMVNLENPEGLPVK	+	2	22	123	86% Complement C3 (*Homo sapiens*)	12	2	14
HGECAVCAHGDLLEGHA	–	2	17	81	–	15	3	18
AHKSRVEAELRSLLAKKFDLGEENFK	+	3	26	83	62% Isoform 3 of Albumin (*Homo sapiens*)	9	2	11
HLTCDELHGDLLEAHGDDR	+	2	19	87	–	11	3	14
MPPHLDQENSESTPADLPSLAA	–	2	22	67	–	14	4	18
RPHSMSALEVDEGSMDPK	+	2	18	43	–	33	11	44

Tryptic peptide is the sequence whose C-terminal amino acid corresponds to R (arginine) or K (lysine); the aliphatic index denotes the relative content of hydrophobic amino acid residues and the volume occupied by side groups of amino acid residues. Proteins with a high aliphatic index (≥70) are thermally stable; Blast–the result of searching for proteins with a matched sequence (Target database: UniProtKB, reference proteomes + Swiss-Prot, No restriction by taxonomy, Reviewed Swiss Prot).

Another important aspect of using *de novo* protein sequencing tools is searching for putative proteoforms caused by single amino-acid polymorphisms (SAPs), alternative splicing and post-translational modifications. Regardless of polymorphism searching accuracy, the *de novo* tools allow one to generally increase the coverage of identified protein sequences, thus improving analysis validity. Table 5 lists individual examples of proteins for which contigs with inaccurate sequence match (an error or proteoform) were detected, supplementing the identification pattern.

**TABLE 5:** Increasing the protein sequence coverage with putative noncanonical sequences using the *de novo* protein sequencing strategy.

Uniprot ID	Name	Gene	Mw, kDa	Length, aa	Localization	MaxQuant aa (%)	PowerNovo aa (%)	Noncanonical aa (%)	Addition aa (%)
P02768	Albumin	ALB	69	609	extracellular space	565 (93%)	585 (96%)	186 (31%)	0
P01024	Complement C3	C3	187	1,663	extracellular space	1,325 (80%)	1,179 (71%)	107 (6%)	32 (2%)
P02787	Serotransferrin	TF	77	698	extracellular space	477 (68%)	555 (80%)	57 (8%)	4 (1%)
A0M8Q6	Immunoglobulin lambda constant 7	IGLC7	11	106	extracellular space	34 (32%)	81 (76%)	36 (34%)	3 (3%)
P01860	Immunoglobulin heavy constant gamma 3	IGHG3	49	446	extracellular space	111 (25%)	308 (69%)	48 (11%)	0
P00739	Haptoglobin-related protein	HPR	39	348	extracellular space	87 (25%)	203 (58%)	25 (7%)	8 (2%)
A8K2U0	Alpha-2-macroglobulin	A2M	161	1,474	extracellular space	1,065 (72%)	1,038 (70%)	39 (3%)	0
P01857	Immunoglobulin heavy constant gamma 1	IGHG1	44	399	extracellular space	241 (60%)	280 (70%)	38 (10%)	0
P01859	Immunoglobulin heavy constant gamma 2	IGHG2	44	395	extracellular space	192 (49%)	288 (73%)	38 (10%)	0
P02748	Complement C9	C9	63	559	extracellular space	242 (43%)	121 (22%)	17 (3%)	0
P20742	Pregnancy zone protein	PZP	164	1,482	extracellular space	55 (4%)	484 (33%)	39 (3%)	16 (1%)

Length–length of the amino acid sequence of the protein; MaxQuant aa (%) – length of identified peptides and the coverage (%) of protein sequence identifications when performing search using the MaxQuant approach; PN aa (%) – length of identified contigs and the coverage (%) of protein sequence identifications when performing search using the PowerNovo tool for *de novo* protein sequencing; Noncanonical aa (%) – length of putative noncanonical contigs and protein coverage. The list of sequences is provided in Supplementary Table S3; Addition aa (%) – additional protein sequences.

## 4 Discussion

Nowadays, the high prevalence of mental disorders dictates the need to proceed from the currently used subjective evaluation of patient’s clinical symptoms in diagnosis making to searching for circulating blood biomarkers. On the one hand, searching for molecular markers and targets for therapeutics will shed light on the molecular foundations of pathology development, while on the other hand enhancing diagnostic accuracy as well as the efficiency and timeliness of drug therapy. Over the past 15 years, the omics technologies have been widely used in exploratory studies of complex multifactorial diseases, including schizophrenia (Kopylov et al., 2023; Song et al., 2023; Shboul et al., 2024; Su et al., 2023). In our study, we pay attention to the proteomic features of plasma samples collected from schizophrenia patients. We performed an in-depth analysis of the high-resolution mass spectrometric data recorded in the data-dependent acquisition mode. The MS/MS peptide spectra were identified using the MaxQuant algorithm for the library of known protein sequences and the PowerNovo algorithm for *de novo* protein sequencing (i.e., without using sequencing libraries or reference spectra). We have demonstrated that at the level of high-abundance (major) proteins, both strategies show very similar results both for the identified proteome and for sequence coverage. However, the strategies complement each other in the zones of mid- and low-abundance proteins. This comprehensive approach ensures the most complete proteomic coverage of the recorded tandem spectra. We complemented the analysis of 18 group-specific proteins (i.e., proteins identified predominantly in blood samples of schizophrenia patients) with putative proteoforms (namely, phosphorylated forms of nine proteins). So far, very few studies have focused on the role of protein post-translational modifications (PTMs) in schizophrenia patients (Smith and Carregari, 2022). We would like to emphasize that phosphorylation, the most common naturally occurring PTM, may cause local changes near the modification site or global structural changes in a protein, thus modulating its biological function. Phosphorylation is a typical variant of protein activity regulation in the cell cycle in the norm, but can also be involved in signaling disorders in pathology (Nikolsky et al., 2023).

Among group-specific proteins associated with schizophrenia, there is a rather extensive group of proteins involved in functioning of dopaminergic and glutamatergic synapses, angiogenesis and cell migration, protein stability and degradation, as well as a large group of proteins participating in transcriptional regulation. These groups of proteins are being actively discussed in the literature in the context of development of neurological disorders. We summarized the findings obtained for each protein group and supplemented them with the currently available data.

### 4.1 Synapse

**FIGURE 3:**
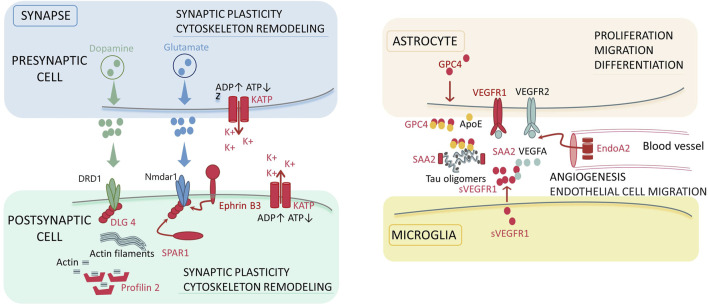
The molecular participants of the synapse and synaptic environment, which can be associated with the development of schizophrenia. Colored zones denote localization of the signaling pathways in the dopaminergic and glutamatergic synapses, astrocyte, microglia, and blood vessel. Red color denotes the molecular factors (proteins) revealed in our study. The key biological processes involving the detected proteins are specified for each localization: synaptic plasticity, cytoskeleton remodeling for synapsis, proliferation, migration and differentiation for astrocyte; angiogenesis and endothelial cell migration for blood vessel. Abbreviations: ADP/ATP–adenosine triphosphate and diphosphate; K_ATP_–ATP-sensitive potassium channels; DRD1 – dopamine receptor D1; Nmdar1 – N-methyl-D-aspartate receptor; DLG4 – discs, large homolog 4; SPAR1 – signal-induced proliferation-associated 1-like protein 1; VEGF (R) – vascular endothelial growth factor (receptor); s–soluble form; EndoA2 – endophilin-A2; GPC4 – glypican-4; SAA2 – serum amyloid A-2 protein; ApoE–apolipoprotein E4.

Synaptic plasticity is mediated by the dynamic localization of proteins to and from synapses (Brigidi et al., 2015). Studying the molecular mechanisms underlying the activity-mediated trafficking of proteins to and from synaptic compartments is crucial for understanding brain functions in the norm and pathology (Brigidi et al., 2015). We pay attention to several proteins engaged in implementation of synaptic plasticity, which may possibly be involved in the pathogenesis of schizophrenia as compensatory mechanisms (Figure 3).

#### 4.1.1 DLG4 and Ephrin-B3

Disks large homolog 4 (DLG4) is a postsynaptic scaffolding protein that plays a critical role in synaptogenesis and synaptic plasticity by providing a platform for the postsynaptic clustering of crucial synaptic proteins. It interacts with the cytoplasmic tail of NMDA receptor subunits and Shaker-type potassium channels. This protein is required for synaptic plasticity associated with NMDA receptor signaling. Overexpression or depletion of DLG4 alters the ratio of excitatory to inhibitory synapses in hippocampal neurons. In the literature, DLG4 is mentioned in the context of the development of neurological disorders. Genetic variants of DLG4 are possibly associated with synaptic dysfunction, which results in DLG4-related synaptopathy; such conditions as neurodevelopmental disorders, intellectual disability (ID), develop of schizophrenia, autism spectrum disorder (ASD) and cerebral visual impairment (Xing et al., 2016; Tokunaga et al., 2024; Bosch et al., 2016; Moutton et al., 2018) are observed in individuals having it.

Ephrin-B3 is another postsynaptic neuron molecule. Upregulated expression or suppression of synthesis of the DLG4 product has a similar effect for ephrin-B3 (Hruska et al., 2015; McClelland et al., 2010). Ephrin-B3 is a transmembrane cell-surface ligand for Eph receptors and is involved in regulation of actin cytoskeleton organization and migration of glutamate receptors (Henderson and Dalva, 2018). The DLG4 and Ephrin-B3 molecules are possibly the components of the same biochemical complex (Hruska et al., 2015; McClelland et al., 2010). In the literature, Ephrin-B3 is reported to be involved in regulation of innate emotional responses and fear accompanying the development of early emotional circuit disruptions in individuals with autism and schizophrenia (Zhu et al., 2016). Ephrin-B3 is a promising drug candidate for managing neurodegenerative diseases or traumatic brain injury (Chumley et al., 2008).

*Signal-induced proliferation-associated 1-like protein 1* (SIPA1L1), or SPAR1, is also a protein engaged in synaptic activity regulation that probably plays a role in maintaining normal neuronal activity. In our study, we pay attention to the putative phosphorylated proteoform at position 111-SEP. The biological role of this protein is not yet entirely clear. Two different mechanisms of SPAR1 function have been discussed in the literature: involvement in actin cytoskeleton reorganization, regulating spine growth and synaptic scaling within the DLG4 complex (PSD95) and NMDA receptor (Matsuura et al., 2022; Pak et al., 2001; Maruoka et al., 2005), as well as G protein-coupled receptor (GPCR) signaling together with partners, spinophilin and neurabin-1 (Matsuura et al., 2021). Findings are also available that SPAR1 is phosphorylated by polo-like kinase 2 (Plk2) and further undergoes degradation via the ubiquitin-proteasome pathway. It has been demonstrated in animal models that in *Sipa1l1* ^−/−^ knockout mice, lack of SPAR1 causes hyperactivity, increased anxiety, learning disabilities, social skills deficits, and susceptibility to epileptic seizures (Pak and Sheng, 2003).

#### 4.1.2 KCNJ11

ATP-sensitive potassium channels (K_ATP_) are widely expressed in metabolically active tissues, including neurons (Zawar et al., 1999). K_ATP_ channels play a crucial role in coupling cell metabolism to electrical activity; they were shown to be involved in several important biological processes such as insulin secretion and synaptic signal transduction (Sun et al., 2008). K_ATP_ channels are present in the brain in the presynaptic and postsynaptic membrane as well as in glial cells. Activation of postsynaptic K_ATP_ channels causes membrane hyperpolarization, which limits neuronal overexcitation; activation of presynaptic channels can directly modulate neurotransmitter release from nerve endings (Sun et al., 2008). Activators of K_ATP_ channels are efficient tools that can regulate cellular excitability and exhibit beneficial effects in patients with pathologic conditions such as ischemia, stroke, and neurodegenerative diseases (Sun et al., 2008; Tai et al., 2003; Wang et al., 2006; Yang et al., 2004).

#### 4.1.3 Profilin-2

The picture of synapse regulators is completed by profilin two protein. Four isoforms are known to exist in mammals; among those, only profilin two is highly expressed in neurons of the central and peripheral nervous system; profilin 1 is expressed ubiquitously; profilins 3 and 4 are expressed in testes (Di Domenico et al., 2021). In our study, we identified a putative phosphorylated proteoform of profilin two at position 92-SEP. This modification has been annotated in the PhosphoSitePlus database (https://www.phosphosite.org/). Profilin two is an actin-binding protein (1:1 stoichiometry) and is needed for actin polymerization in the synapse. It also promotes synaptic vesicle exocytosis and neuronal excitability. The protein is involved in various cellular pathways (Pilo Boyl et al., 2007).

### 4.2 Angiogenesis and endothelial cell migration

*Endophilin -A2* (EndoA2) proteform at position 75-SEP, which has been annotated in the PhosphoSitePlus database, was identified in our study. Endophilins are multidomain cytoplasmic proteins involved in formation and degradation of endocytic vesicles (The structure and function of endophilin proteins, 2010). EndoA2 and VEGFR2/VEGFA control migration, proliferation, and survival of endothelial cells (Genet et al., 2019). The molecular mechanisms still remain to be elucidated. However, *EndoA2* knockout mice have defective postnatal angiogenesis. EndoA2 deficiency reduces VEGFR2 internalization (Genet et al., 2019).

#### 4.2.1 VEGF receptor 1

Vascular endothelial growth factors (VEGF), which were originally believed to have an impact exclusively on the vascular system, exhibit trophic effects on neurons during ontogenesis and adulthood. Today, VEGF receptors 1 and two are also known to be expressed in neurons (Okabe et al., 2020; Wittko-Schneider et al., 2013; Selvaraj et al., 2015). VEGFR1 is presumably a receptor promoting glial development and survival, being involved in neuroprotection. Furthermore, alternative splicing produces a soluble truncated form of sVEGFR1, which competes for the VEGFA ligand and regulates the signaling pathway mediated by the membrane-bound form of the receptor via the negative coupling mechanism (Wittko-Schneider et al., 2013). The VEGF system is a promising therapeutic target for treating brain pathologies (Wittko-Schneider et al., 2013). The product of VEGFR1 expression plays a role in the inflammatory responses (Shibuya, 2006; Sarabipour et al., 2024) accompanying some mental disorders such as depression and schizophrenia. The role played by VEGFR1 in vascular health may also indirectly affect brain function, since proper blood flow is critical for maintaining cognitive function (Khan et al., 2014).

### 4.3 Protein stability and degradation

*Glypican 4* (GPC-4), a protein secreted by astrocytes, further binds to apolipoprotein E4 (APOE4). The resulting complex causes tau hyperphosphorylation and aggregation (Frontiers Editorial, 2019) (Figure 3). Tau aggregation is known to be a major factor in neurodegeneration and behavioral disorders in patients with tauopathies, including Alzheimer’s disease (Saroja et al., 2022).

*Serum protein amyloid A2* (SAA2) belongs to the serum amyloid A family of apolipoproteins. This protein is highly expressed during inflammation and tissue injury. Studies have demonstrated that SAA2 is associated with several chronic inflammatory diseases, including Alzheimer’s disease. In particular, SAA proteins play a regulatory role in tau phosphorylation, which is a hallmark of Alzheimer’s disease (Liu et al., 2016).

*Leucine-rich repeats and immunoglobulin-like domains 1* (LRIG1) are the transmembrane protein whose extracellular domain contains 15 leucine-rich repeats (LRRs). LRIG1 is involved in modulation of signaling from growth factors and acts as a tumor suppressor (Saroja et al., 2022; Liu et al., 2016). A tentative mechanism is that LRIG1 binds to the epidermal growth factor receptor (EGFR) ectodomain and triggers EGFR ubiquitination followed by lysosomal degradation. All of these factors reduce the levels of cell surface receptors (Wang et al., 2013). No association between the protein and cognitive or behavioral impairment has been revealed yet.

#### 4.3.1 UBL conjugation pathway

Proteins engaged in the ubiquitin-like protein (UBL) conjugation pathway are integral components of cellular protein homeostasis (Bedford et al., 2011). Attachment of UBLs to proteins regulates numerous cellular processes, including transcription, cell cycle, stress responses, DNA repair, apoptosis, immune responses, and autophagy (Streich and Lima, 2014). The functionally distinct conjugation pathways typically involve three types of proteins: (Howes et al., 2024): E1 Ubl activating enzymes, and these very components are responsible for specificity of each cascade; (Schizophrenia, 2022) E2 Ubl transfer proteins; and (Solmi et al., 2023) E3 Ubl ligases. In our study, we identified the following components: E2 IFT74; E3 HERC1, ASB7, and USP9Y. In the literature, dysfunction of the UBL conjugation pathway has been linked to the development of a broad range of diseases, including schizophrenia (Rubio et al., 2013). Interestingly, the *HERC1* gene is associated with various mental disorders. Research suggests that mutations in the *HERC1* gene may contribute to developmental abnormalities of the nervous system and development of mental illnesses such as autism spectrum disorder, depression, and schizophrenia (Vogel et al., 2023). However, the role of the products of other genes in the pathogenesis of neurological diseases still needs to be elucidated.

#### 4.3.2 Zinc finger proteins and transcriptional regulators

Zinc finger proteins (ZNFs) are the most abundant proteins in the eukaryotic genomes. They share such feature as presence of zinc finger domains that can selectively bind to a certain DNA or RNA. Zinc finger proteins are involved in regulation of gene expression at both the transcriptional and translational levels. Numerous studies have demonstrated that ZNF ensembles are associated with neurological diseases. In this work, we identified ZNF880, ZNF 347, and ZNF48, as well as TRERF1 and NPAT, as transcriptional regulation factors. No association with psychiatric disorders was found for most protein products of these genes. However, it is known that TRERF1, transcriptional regulating factor 1, is involved in regulation of expression of the cytochrome P450 11A1 (CYP11A1). This enzyme catalyzes the synthesis of pregnenolone substrate for all the known steroids (Moschny et al., 2020). TRERF1 can possibly be associated with the response to electroconvulsive therapy in patients with major depressive disorder and plays a role in the mechanisms underlying some mental illnesses, including depression. Another participant, NPAT, is probably involved in neurodevelopmental disorders such as intellectual disability (Sang et al., 2022).

## 5 Conclusion

Schizophrenia remains among the most complex and understudied diseases in psychiatry that severely affects millions of people worldwide. This disease is often diagnosed late, when symptoms become apparent and difficult to reverse. Research into its molecular mechanisms offers opportunities for finding novel biomarkers and targets for drug therapy. Our study focuses on proteomic analysis using the MaxQuant software for the library of known protein sequences and the PowerNovo tool for *de novo* protein sequencing. We demonstrated that both strategies support the results obtained by each other in the case of high-abundance circulating proteins and complement each other in the case of mid- and low-abundance proteins. We focus on group-specific proteins (n = 18) and tentative phosphorylated proteoforms (n = 9), which were detected in the samples collected from schizophrenia patients. These proteins are involved in synaptic plasticity, angiogenesis, transcriptional regulation, protein stabilization and degradation. The identified proteins are mentioned in the literature in the context of the development of neurological and psychiatric disorders. An important result was the identification of contigs: peptide/protein sequences that are also group-specific for schizophrenia patients. These contigs have not been annotated yet in protein sequence libraries, and no orthologs have been found for them.

Further research should focus on in-depth investigation of proteins engaged in synaptic processes, angiogenesis, protein degradation, and transcriptional regulation.

## Data Availability

The datasets presented in this study can be found in online repositories. The names of the repository/repositories and accession number(s) can be found in the article/Supplementary Material.
